# tDRmapper: challenges and solutions to mapping, naming, and quantifying tRNA-derived RNAs from human small RNA-sequencing data

**DOI:** 10.1186/s12859-015-0800-0

**Published:** 2015-11-04

**Authors:** Sara R. Selitsky, Praveen Sethupathy

**Affiliations:** 1 Bioinformatics and Computational Biology Curriculum, University of North Carolina, Chapel Hill, NC USA; 2 Departments of Genetics, University of North Carolina, Chapel Hill, NC USA; 3 Microbiology & Immunology, University of North Carolina, Chapel Hill, NC USA; 4 Lineberger Comprehensive Cancer Center, University of North Carolina, Chapel Hill, NC USA

**Keywords:** tRNA, tDR, Sequencing, RNA modifications, Bioinformatics

## Abstract

**Background:**

Small RNA-sequencing has revealed the diversity and high abundance of small RNAs derived from tRNAs, referred to as tRNA-derived RNAs. However, at present, there is no standardized nomenclature and there are no methods for accurate annotation and quantification of these small RNAs. tRNA-derived RNAs have unique features that limit the utility of conventional alignment tools and quantification methods.

**Results:**

We describe here the challenges of mapping, naming, and quantifying tRNA-derived RNAs and present a novel method that addresses them, called *tDRmapper*. We then use *tDRmapper* to perform a comparative analysis of tRNA-derived RNA profiles across different human cell types and diseases. We found that (1) tRNA-derived RNA profiles can differ dramatically across different cell types and disease states, (2) that positions and types of chemical modifications of tRNA-derived RNAs vary by cell type and disease, and (3) that entirely different tRNA-derived RNA species can be produced from the same parental tRNA depending on the cell type.

**Conclusion:**

*tDRmapper*not only provides a standardized nomenclature and quantification scheme, but also includes graphical visualization that facilitates the discovery of novel tRNA and tRNA-derived RNA biology.

## Background

Transfer RNAs (tRNAs) are non-coding RNAs that deliver amino acids to ribosomes during translation. tRNA-derived RNAs (tDRs) are small RNAs that are enzymatically processed from either nascent pre-tRNA transcripts or mature tRNAs [1]. Their regulated biogenesis and well-defined 5′ and 3′ ends indicate that they are not products of tRNA degradation [2]. tDRs are generated in organisms from all domains of life [3]. They are derived from most tRNA genes and produced in varying abundance, in a variety of different sizes, and from different regions of the tRNA. Several functions have been attributed to tDRs such as post-transcriptional [4, 5] and translational repression [6], stress granule formation [7], and protection from apoptosis [8]; however, all of these have been in the context of cell culture. The role of tDRs in human health is only now starting to emerge. tDRs may play a role in neurodegeneration [9], cancer [5, 10], as well as immune modulation [11], and we previously showed that tDRs are significantly increased in the liver tissue of patients with chronic viral hepatitis and decreased in liver cancer [2].

Despite the potential biomedical significance of tDRs, the field is lagging behind other small RNA fields in terms of genomic annotation and strategies for quantification from small RNA-sequencing (small RNA-seq) data. This is due in large part to: (a) the unique computational challenges of mapping tDRs from small RNA-seq data and (b) the lack of a standardized nomenclature for tDRs.

- While small RNA-seq has enabled the discovery of tDRs, these small RNAs are difficult to map accurately for at least three reasons:
  1. Exact copies of tRNA genes are present in numerous locations throughout the human genome, and annotation of tRNAs in the human genome is still incomplete. This means that small RNA-seq reads corresponding to tRNA-derived RNAs can map with equal fidelity to numerous locations throughout the genome (multi-mapping), which leads to ambiguity about the precise origin of the tRNA-derived RNA.
  2. tDRs are derived from both the nascent pre-tRNAs and the processed, mature tRNAs. The maturation process of eukaryotic tRNAs includes several steps, such as the removal of 5′ leader sequence and 3′ trailer sequence, the addition of a non-templated “CCA” to the 3′ end, and the excision of introns. These changes during maturation need to be accounted for when mapping tRNA-derived RNA reads. For example, spliced reads (those derived from the sequence flanking the spliced intron) or reads that contain a non-templated 3′-end “CCA” will not map to the genome.
  3. tRNAs are subject to extensive chemical modifications at specific nucleotide positions during maturation [12]. As a result, tDRs most likely harbor these modifications, which can lead to errors during cDNA synthesis due to reverse transcriptase pausing and mis-incorporation of nucleosides [13]. These errors manifest in small RNA-seq reads as mismatches and deletions relative to the reference tRNA sequence. These mismatches/deletions will be referred to as “error type.”
- There is no standardized nomenclature for tDRs. tDRs are produced in a variety of different sizes, from a variety of different tRNAs, and from a variety of different locations within the tRNAs, all of which present challenges for a coherent naming system. A standard naming scheme is critical to facilitate future research. For example, it is at present extremely difficult to use published studies to define the bio-distribution of specific tDRs (the tissues and conditions in which specific tDRs are expressed) because the same or similar tDR is often referred to by completely different names (and in some cases a name is not given at all).

In this study we introduce a tool designed to address the challenges of mapping, naming, and quantifying tDRs, called *tDRmapper. tDRmapper* was designed specifically for human small RNA-seq data (single-end, 50x) generated on the Illumina sequencing platform using cDNA libraries that were prepared using the Illumina TruSeq protocol. We used *tDRmapper* to analyze publically available small RNA-seq datasets (total *n* = 45) from four categories of cell types/tissues. These analyses helped shape the final version of the tool and also led to the discovery of new types of tDR species as well as novel insights about potentially varying patterns of tRNA modifications in different human tissues and diseases.

## Results and discussion

*tDRmapper* aligns small RNA-seq reads to tRNA sequences, allowing for and quantifying mismatches and deletions. *tDRmapper* was written with its own string matching alignment scheme and does not use a previously developed aligner, such as Bowtie 2 [14]. While Bowtie 2 does allow for mismatches and deletions, it was not designed with unique features of tRNAs and pre-tRNAs in mind. *tDRmapper* is specifically designed for mapping reads to tRNAs, which have several unique features that are described below. Also, while methods for assessing RNA modifications from small RNA-seq data have been previously developed, such as HAMR [13], they do not quantify tDR species. *tDRmapper* defines and quantifies specific tDRs and automatically generates visualization of both pre- and mature tDR profiles.

### Small RNA-seq datasets from four categories of human cell types/tissues

We analyzed a diverse array of human small RNA-seq datasets to guide tool development and to ensure that *tDRmapper* is not biased to one specific tissue or disease. We also used some of the results from these analyses as examples to illustrate the importance of each step in *tDRmapper*.

**Fig. 1:**
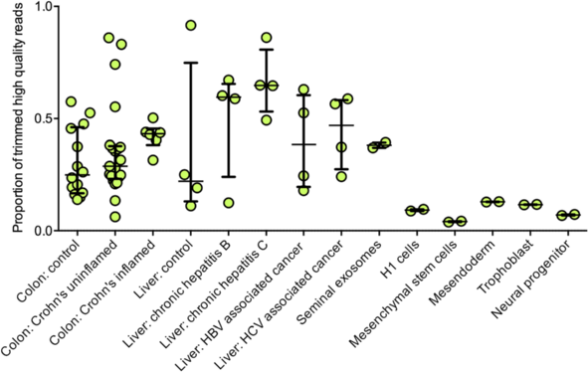
Relative abundance of all tRNA-derived RNAs across four categories of human cell types/tissues. Proportion of trimmed and filtered reads that map to tRNAs in primary colon tissue (control *n* = 13; Crohn’s disease uninflamed tissue, *n* = 21; Crohn’s disease inflamed tissue, *n* = 6), primary liver tissue (control, *n* = 4; chronic hepatitis B non-cancer tissue, *n* = 4; chronic hepatitis C non-cancer tissue, *n* = 4; chronic hepatitis B associated cancer tissue, *n* = 4; chronic hepatitis C associated cancer tissue, *n* = 4), the non-sperm fraction of seminal fluid (*n* = 2), and NIH roadmap H1 and H1-derived cells (H1 cells with no treatment, *n* = 2; H1-derived trophoblasts, *n* = 2; H1-derived mesendoderm, *n* = 2; H1-derived neuronal progenitor cells, *n* = 2; H1-derived mesenchymal stem cells, *n* = 2). *Black lines* show the median and interquartile range

Previously, we reported that tDRs are highly abundant in liver tissue and both the total tDR abundance and the relative expression of individual tDRs were associated with chronic hepatitis B, chronic hepatitis C, and hepatocellular carcinoma [2]. In this study, the analysis of the different categories of cell types/tissues revealed that the high abundance of tDRs is not specific to liver disease, but rather tDRs are present in high abundance in other human tissues and fluids as well (Fig. 1). The four categories of cell types/tissues are described below:

#### Primary liver tissue – Chronic viral hepatitis and liver cancer [2]

Hepatitis B and hepatitis C viruses both infect the liver, leading to chronic liver infection and cancer. Both viruses together are responsible for ~80 % of the world’s liver cancer cases. Liver tissue was collected during surgical resection in Kanazawa, Japan and small RNA-seq was performed on the RNA isolated from matched malignant and non-malignant tissue from patients with advanced chronic hepatitis B (*n* = 4) or chronic hepatitis C (*n* = 4), as well as from liver tissue from uninfected individuals (*n* = 4) undergoing liver surgery due to metastatic colon cancer (GSE57381).

#### Primary colon tissue – Crohn’s disease [15]

Crohn’s disease is a chronic inflammatory condition of the gastrointestinal tract. These datasets were generated from a study that performed small RNA-seq on primary colon tissue from subjects with Crohn’s disease, including both the uninflamed (*n* = 21) and inflamed (*n* = 6) components, as well as colon tissue from individuals without Crohn’s disease (*n* = 13). The small RNA-seq was performed in the same sequencing facility as the liver samples described above (GSE66209).

#### Seminal fluid – Prostate cancer [16]

The non-sperm fractions of seminal fluid can be used to screen for biomarkers of prostate cancer. These datasets were generated from a study that performed small RNA-seq on a pooled sample of seminal fluid RNA from men with prostate cancer (*n* = 6) and a pooled sample of seminal fluid RNA from men without prostate cancer (*n* = 6) (GSE56686).

#### Stem and progenitor cells – Phases of embryonic development

The University of California at San Diego, through the NIH Roadmap Epigenomics Project, generated small RNA-seq data for ten samples (GSE16256): H1 cells (human embryonic stem cells) with no treatment (*n* = 2), H1 cells treated with BMP4 for differentiation to trophoblasts (*n* = 2) and mesendoderm (*n* = 2), H1-derived neuronal progenitor cells (*n* = 2), and H1-derived mesenchymal stem cells (*n* = 2).

### Details of tDRmapper

**Fig. 2:**
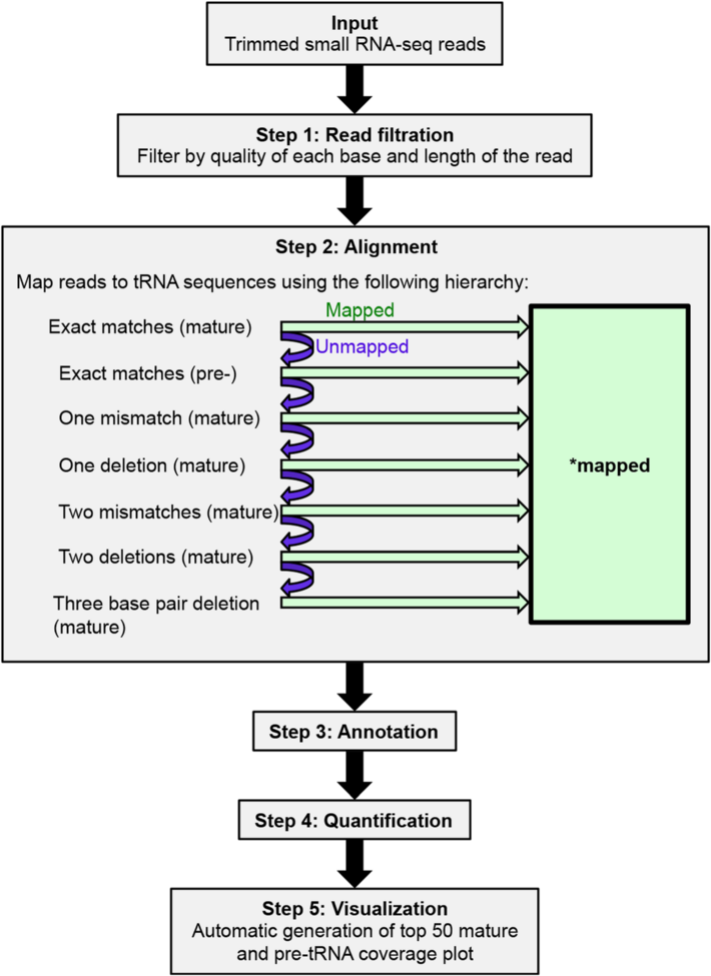
Schematic of *tDRmapper*. The input into *tDRmapper* is trimmed small RNA-seq reads. (Step 1) Reads are discarded if quality <28 at any position, length <14 or >41, or if the sequence does not occur >100 times in the FASTQ file. (Step 2) Reads are aligned according to a specific “error type hierarchy.” First reads are aligned, allowing for exact matches only to mature tRNA sequences, then reads that do not map are aligned allowing for exact matches to pre-tRNA sequences, and then reads that do not map are aligned allowing for one mismatch, then one deletion, two mismatches, two deletions, and then a three base pair deletion to mature tRNA sequences. (Step 3) tDRs are annotated based on size and location within either pre-tRNA or mature tRNA. (Step 4) tDRs are quantified based on two features, the fraction of reads aligning to the parent tRNA and the maximum coverage across all positions of the tRNA. (Step 5) tDRs are visualized as color-coded coverage maps

We developed the first publically available tool, called *tDRmapper*, for mapping, naming, and quantification of tDRs (Fig. 2). Each step of *tDRmapper* is described below:

#### Step 1: Sequencing reads are filtered for quality

*tDRmapper* takes as input adapter-trimmed small RNA sequencing reads in FASTQ format. The program then discards the following types of reads:

1. If the Phred +33 quality score drops below 28 at any individual position along the length of the read. This filter is implemented in order to increase the likelihood that mismatches/deletions in the reads with respect to the reference sequence are biological and not due to errors of the sequencing machine. The score threshold is very conservative, so the mismatch/deletion (“error type”) calls are high confidence.
2. If the read is smaller than 14 nucleotides or larger than 40. *tDRmapper* was designed for single-end 50x small RNA-seq data. Based on our adapter trimming strategy, successfully trimmed reads are <41 nts. The lower bound (14 nts) is especially critical for mitigating mapping ambiguity (the smaller the read the more locations it can map to just by chance, especially when allowing for mismatches and deletions).
3. If the read does not occur in the FASTQ file at least 100 times. This threshold was set based on our observation that reads with count less than 100 appeared to contribute more noise (small degraded fragments) than signal (true tDRs). For datasets with vastly different total number of reads, it may be advisable to change the threshold into a percentage of total reads rather than a number of reads.

#### Step 2: Filtered reads are aligned to mature and pre-tRNAs following an “error type” hierarchy

##### Alignment to tRNA sequences

Reads are aligned to mature and pre-tRNA sequences and not the genome for four reasons:

1. The total number of human tRNA genes is not known and tRNA annotation across the human genome remains an open area of research. Therefore, some reads that map to annotated tRNAs will also map equally well to other regions of the genome that may also be tRNAs, but are currently unannotated. Mapping the small RNA-seq reads to known tRNA sequences avoids this ambiguity. Of the 615 currently annotated human tRNA genes in The Genomic tRNA Database [17] (gtRNAdb), 239 have one or more annotated identical copies across the genome. For example, one of the tRNA-Histidine genes has nine exact copies across the human genome and one of the tRNA-Aspartate genes has 11. Mapping reads that are derived from multi-copy tRNA genes to the whole genome would complicate tDR quantification. Our solution is to aggregate the human tRNA genes that have identical sequences (both pre- and mature sequences) into “families” and give those “families” one name (FASTA included with *tDRmapper*).
2. Many tDRs are thought the be derived from processed, mature tRNAs. As stated previously, tRNAs undergo specific changes during maturation and as a result, the sequences of mature tRNAs do not reflect the genomic template from which they are produced. These changes during maturation need to be accounted for when mapping tRNA-derived RNA reads.
3. Aligning to annotated tRNA sequences instead of the genome has the added benefit of a much smaller search space, which means that mismatches and deletions can be accounted for in a much more computationally tractable manner.

tRNA sequences were downloaded from gtRNAdb. To generate the mature tRNA libraries, we removed the predicted intronic sequences (if present) and added an additional 3′ terminal “CCA” to each tRNA. To generate the pre-tRNA libraries, we included 40 nucleotides of flanking genomic sequence on either side of the original tRNA sequence.

##### Error type

Small RNA-seq reads are aligned with an “error type” hierarchy (Fig. 2) and each “error type” is annotated and quantified. The reads are first mapped to mature tRNA sequences allowing for exact matches only. The reads that do not align are then funneled to the next step, where the reads are mapped to pre-tRNA sequences allowing for exact matches only. The reads that do not align exactly to either the mature tRNA or the pre-tRNA are then mapped to mature tRNA sequences, allowing first for one mismatch, then one deletion, two mismatches, two deletions, and lastly a three base pair deletion. *tDRmapper* is written in a modular fashion, meaning each “error type” alignment is written in a separate script, enabling the user to easily switch the sequential order in which the algorithm searches for different “error types.” This may be useful if, for example, there emerges a compelling biological reason to prioritize one deletion over one mismatch or to speed up the program by not allowing two mismatches.

We allow for and annotate “error types” in reads mapping to mature tRNA sequences because mature eukaryotic tRNAs have on average 17 chemical modifications [12]. Some of these modifications can lead to pausing and mis-incorporation of nucleosides, which manifest as mismatches and deletions in the small RNA-seq reads [13, 18]. We only allow for mismatches and deletions when mapping to mature tRNAs, and not pre-tRNAs, because we assume that only mature tRNAs are subject to extensive chemical modifications. If future research overturns this assumption, then the algorithm can be amended accordingly in a relatively straight-forward manner. (We note here that some modifications could also lead to reverse transcriptase aborts, which would result in an under-representation of certain types of reads in small RNA-seq data. While this is an important issue, it is best addressed by experimental solutions, so we do not discuss this in *tDRmapper*).

**Fig. 3:**
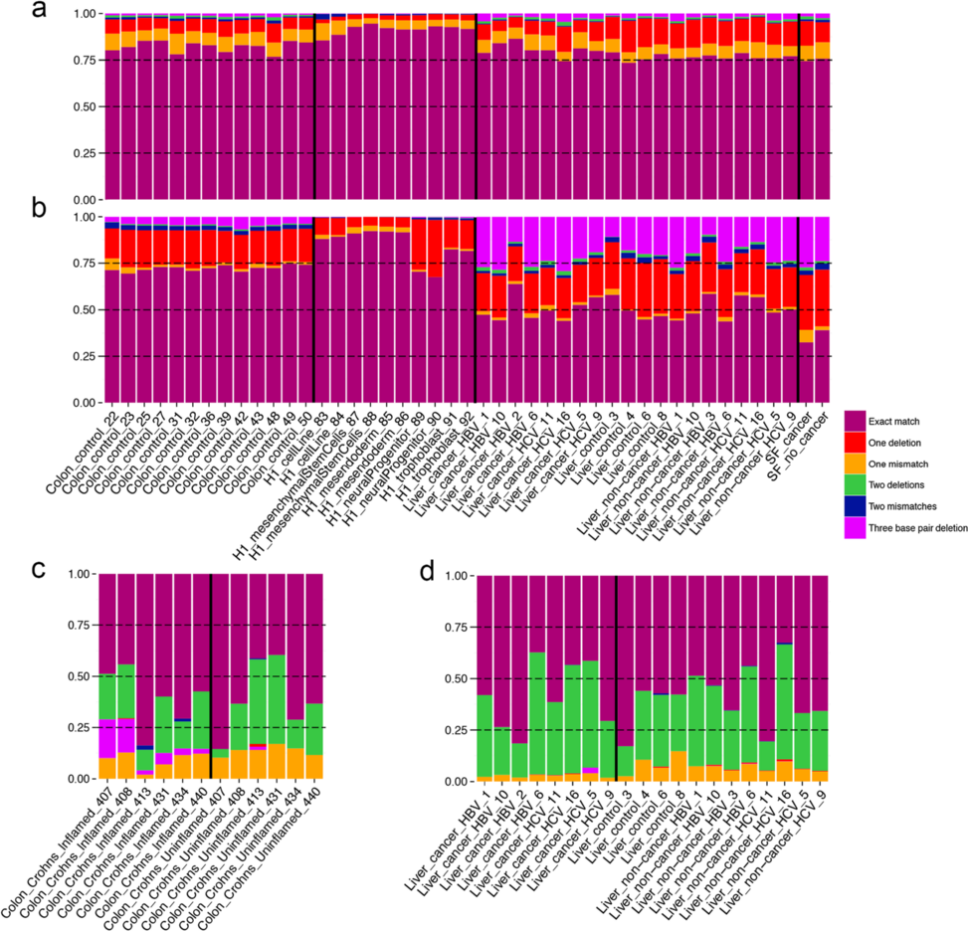
Proportion of “error types” for specific tRNA-derived RNAs across different tissues and diseases. **a**-**d** Relative proportion of “error types” of reads mapping to **a** all tRNAs, **b** tRNA-Glu-CTC-1-7, **c** tRNA-Pro-CGG-1-3, **d** tRNA-Gly-GCC-1-5

tRNA modifications have been associated with disease [19]. We sought to annotate the locations and “error types” across different tissues and diseases, as these “error types” may represent sites of chemical modification. Figure 3a shows the “error type” proportion across all tRNAs for all of the datasets analyzed. Overall, most reads align to tRNAs exactly; however, there are some tRNAs that have a higher abundance of mismatches and deletions than others, and also exhibit substantive variation in “error types” across tissues (Fig. 3b). For example, in the liver and seminal fluid samples, ~20 % of the reads that mapped to tRNA-Glu-CTC-1-7 only aligned after allowing for a three base pair deletion, while in colon tissue and H1 cells, this accounted only for ~4 % and <0.5 %, respectively (Fig. 3b). This example demonstrates tissue-specific profiles of “error types” and the importance of allowing for deletions and mismatches in tDR alignment, since without this feature, ~50 % of reads aligning to tRNA-Glu-CTC-7-1 in the liver tissue and seminal fluid samples would be discarded.

We also found that “error type” profiles can vary by disease within the same tissue for specific tDRs (Fig. 3c, d). For example, on average, 8 % of reads that correspond to tRNA-Pro-CGG-1-3 in the inflamed Crohn’s tissue only mapped after allowing for a three base pair deletion, whereas in 5/6 of the matched uninflamed tissue, no reads mapped after allowing for a three base pair deletion (Fig. 3c). Also, reads that mapped to tRNA-Gly-GCC-1-5 had a significantly (*P* = 0.001) higher incidence of one mismatch in the non-cancer liver samples compared to the matched cancer samples (Fig. 3d).

#### Step 3: Aligned reads are named based on read size and location of derivation from mature or pre-tRNAs

**Fig. 4:**
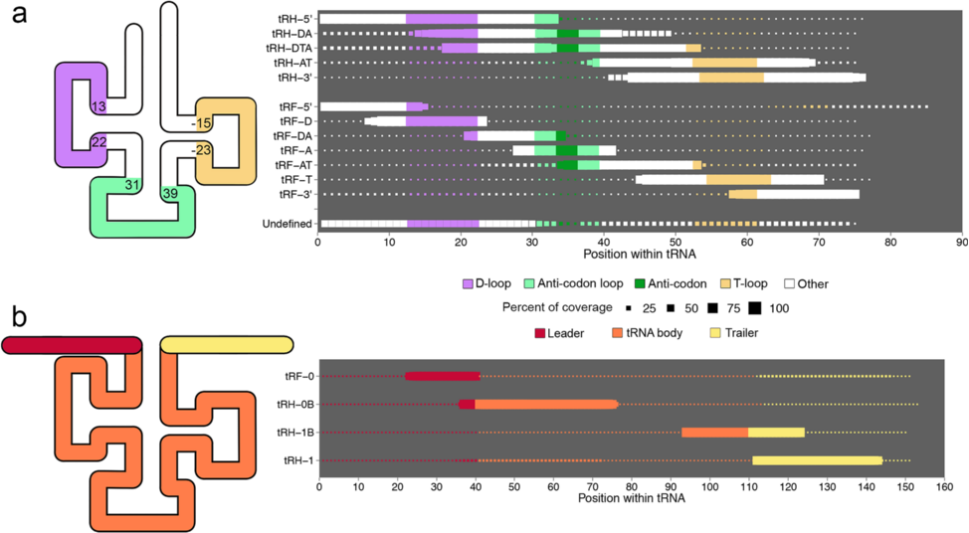
Diagrammatic representation of the naming scheme for tRNA-derived RNAs. **a** & **b** Left panel: color-coded structure of tRNAs to illustrate how tDRs are named. Colors in panel correspond to coverage maps in right panel. Right panel: Example tDRs identified in the datasets analyzed are shown. Size of dot represents percent of reads mapping at each position within each tRNA shown. **a** Naming scheme of tDRs derived from mature tRNAs. Numbers correspond to the “generalized” start and stop positions for each loop. Purple represents the D-loop, “D”; green represents the anti-codon loop, “A”; dark green represents anti-codon triplet; and yellow represents T-loop, “T”. **b** Naming scheme of tDRs derived from pre-tRNAs. Red represents the leader sequence, “0”; orange represents the sequence of the tRNA body,“B”; and yellow represents the trailer sequence, “1”

One of the primary challenges in the tDR field is that there is no standardized naming system. We propose a nomenclature that is both concise and informative (Fig. 4). *tDRmapper* will assign to each tDR a name that has three components:

1. The first component of the tDR name indicates the parent tRNA “family” from which the tDR is derived.
  - For the mature tRNA sequences, the “family” names consist of four parts, “W-X-Y-Z”, where W is the tRNA amino acid (sometimes preceded by “nmt-tRNA,” which means “nuclear encoded mitochondrial tRNA”), X is the anti-codon, Y is a unique identifier for each tRNA family, and Z is the number of mature tRNA genes with the identical sequence. For example, Asp-GTC-2-11 represents a mature tRNA “family” sequence that could be derived from 11 different tRNA genes, has a unique ID, “2”, bears the GTC anti-codon, and associates with Aspartate. This naming scheme builds on the nomenclature for tRNAs used by gtRNAdb.
  - For the pre-tRNA sequences, the “family” names consist of 6 parts, “pre-W-X-Y-Z.n”. This reflects the same naming scheme described above in (a), but with the prefix “pre” and the suffix “.n”. The “.n” refers to the number of pre-tRNAs from the W-X-Y mature tRNA family that have the identical pre-tRNA sequence. From among those with the identical pre-tRNA sequence, the largest “Z” is chosen for the “family” name. For example, the pre-Gly-TCC-2-5.4 sequence accounts for four pre-tRNAs with identical sequence. The family members included under this name are pre-Gly-TCC-2-2, pre-Gly-TCC-2-3, pre-Gly-TCC-2-4, and pre-Gly-TCC-2-5.
2. The second component of the tDR name indicates the size of the tDR. *tDRmapper* determines the primary tDR associated with a parent tRNA by counting the number of positions in the tRNA that have >50 % coverage (where coverage at a position is defined as the percent of all reads mapping to the tRNA at that position). If the primary tDR sequence is <41 nts and = >28 nts, it is defined as a tRNA-half (tRH), and if it is >14 nts and <28 nts, it is defined as a tRNA-fragment (tRF).
3. The last component of the tDR name indicates the region in the mature or pre-tRNA from which the read is derived.
  - For the tDRs derived from mature tRNAs (Fig. 4a), we use a generalized tRNA secondary structure (Methods). First we determine if a read is derived from the 5′ or 3′ end of the tRNA. If not, then we assess whether it overlaps the D-loop, anti-codon loop, or T-loop by at least one nucleotide. Details are provided below:
    i. “5’” is added as a suffix to the tDR name if the coverage is >50 % at position +1 of the tRNA (first nucleotide at the 5′-end).
    ii. “3’” is added as a suffix to the tDR name if the coverage is >50 % at position −7 of the tRNA (7 nucleotides from the 3′ end). Position −7 was used instead of position −1 due to the tapering signal (graded reduction in coverage) observed at the 3′ ends of some tDRs.
    iii. “D”, denoting the D-loop, is added as a suffix to the tDR name if the coverage is >50 % at any position between 13 and 22, and is not >50 % at position 1.
    iv. “A”, denoting the anti-codon loop, is added as a suffix to the tDR name if the coverage is >50 % at any position between 31 and 39, and not >50 % at position 1 or position −7.
    v. “T”, denoting the T-loop, is added as a suffix to the tDR name if the coverage is >50 % at any position between −23 and −15, and not >50 % at position −7. The T-loop is denoted using positions determined from the 3′ end (as opposed to the D-loop and anti-codon loop which are denoted using positions determined from the 5′-end – see [iii] and [iv] above) due to variability in the size of the region between the anti-codon loop and the T-loop (which contains what is known as the “variable loop”).
  - For the tDRs derived from pre-tRNAs, we annotate where in relation to the header, trailer, and tRNA “body” the tDR is derived (where “body” is defined as the full-length tRNA sequence provided by gtRNAdb). Details are provided below (Fig. 4b):
    i. “0” is added as a suffix to the tDR name if the coverage is >50 % at one or more nucleotides 5′ of the tRNA body, the leader sequence.
    ii. “1” is added as a suffix to the tDR name if the coverage is >50 % at one or more nucleotides 3′ of the tRNA body, the trailer sequence. This annotation is consistent with the already described “tRF-1 series” [20], which are tRFs derived exclusively from trailer sequences.
    iii. “B”, denoting tRNA “body”, is added as a suffix to the tDR name (after either a “0” or a “1”) if the coverage is >50 % at any position in the body of the tRNA.

Finally, a primary tDR is only named in the manner described above if at least one position of the tDR has coverage >66 % (that is, more than 2/3 of the total reads mapping to the tRNA). Otherwise the name given is “undefined” (Fig. 4a). The goal of *tDRmapper* is to annotate the clearly dominant tDRs. A threshold lower than 66 % would lead to less confidence in the dominance of a particular tDR. For example, consider a tDR that has a maximal coverage of 52 % and another that has a maximal coverage of 48 %. Technically, the former is the dominant tDR; however, the two tDRs are close enough in representation that the differences in coverage could be due to technical reasons, such as variable read mapping efficiencies. Therefore, in such cases, we take a conservative approach and deem the dominant tDR to be ambiguous. The stringent threshold of 66 % is intended to ensure that a primary tDR is identified only when it represents the vast majority of the reads mapping to the parent tRNA.

#### Step 4: tDRs are quantified based on two features, the fraction of reads aligning to the parent tRNA and the maximum coverage across all positions of the tRNA

The tDRs are quantified using the following equation:

$$ Relative\  abundance=\left(\left(\frac{\#\  of\  reads\  mapped\ to\  the\  tRNA}{\#\  of\  reads\  mapped\ to\  all\  tRNA}\right)\times highest\  prop.\kern0.5em  of\  coverage\  of\  tRNA\right)\times 100 $$

**Fig. 5:**
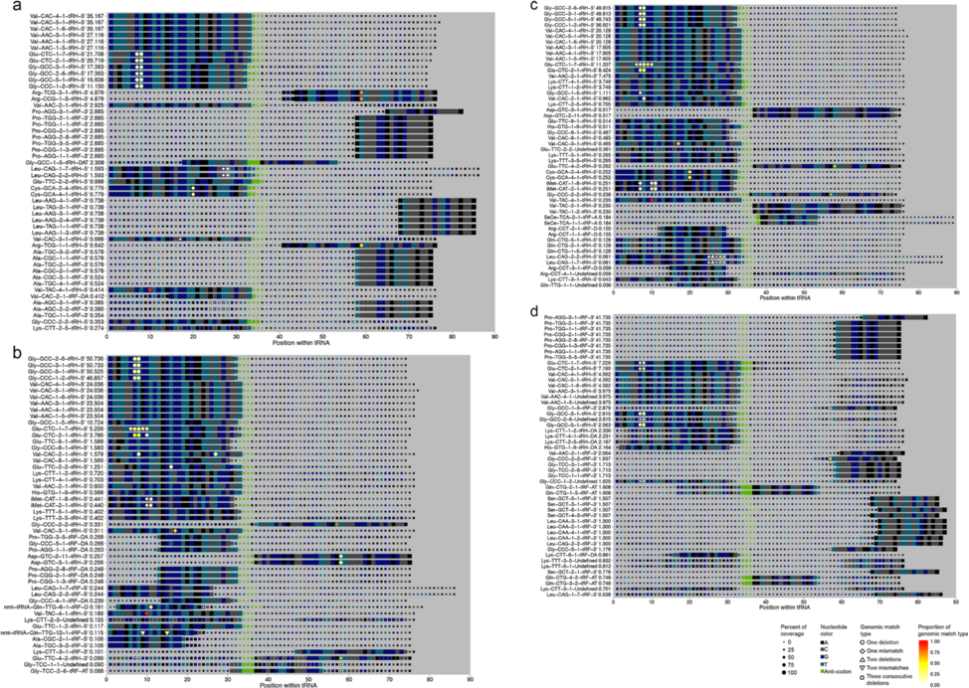
Examples of mature tRNA coverage maps from each category of human cell types/tissues. **a**-**d** Size of dot represents percent of reads mapping at each position within each tRNA. The color represents each individual nucleotide or the anti-codon positions. The dots overlaid in a variety of shapes represent each “error type” and the color of these shapes represents the proportion of each “error type” at each position. Coverage map for (**a**) primary colon tissue from control subject #31, **b** the non-sperm fraction of seminal fluid from subjects with prostate cancer, **c** primary non-cancerous liver tissue from subject #6 with chronic hepatitis B, and **d** H1-derived neural progenitor cells

For example, the relative abundance of Val-CAC-1-6 is 4.6 % in the neural progenitor cells (Fig. 5d). In this sample, there are 45,347 reads mapping to Val-CAC-1-6; 679,288 reads mapping to all the tRNAs; and the highest proportion of coverage across the tRNA-Val-CAC-1-6 is 0.69, meaning the area with the highest read depth across tRNA-Val-CAC-1-6 accounts for 69 % of the reads. By multiplying the total number of reads mapping to a parent tRNA by the maximum proportion of coverage across all base positions in the tRNA, we are computing the fraction of total reads mapping to the parent tRNA that include the most well represented position in the tRNA. The primary tDR spans this position; therefore, this quantification scheme provides a ceiling on the fraction of reads that likely correspond to the primary tDR. In some instances, this approach may overestimate the relative abundance of a primary tDR. In the future, other quantification strategies can also be considered.

Also, every instance a read maps to a tRNA it is counted once. For example, in the control colon patient #30, the read “GTTTCCGTAGTGTAGTGGTTATCACGTTCGCCT” occurs 1,415,079 times in the FASTQ file, maps to six different tRNA-Val “families,” and therefore is quantified as 1,415,079 for each of the families.

There are two reasons for this quantification method:

1. Small RNA-seq reads will often map with equal fidelity to several tRNA families. A common strategy for handling multi-mapping is fractional assignment of reads. Fractional assignment in its most simplistic form divides the read count by the number of locations to which the read aligns. One goal of fractional mapping is to capture all locations at which transcription could be occurring. But, in this scheme, tDRs that derive from tRNAs with many copies throughout the genome will be artificially under-quantified. Since the goal of *tDRmapper* is to quantify the total abundance of tDRs, not gain information about transcriptional regulation of tDRs, we do not use fractional mapping.
2. This quantification method has high resolution since each primary tDR from each parent tRNA family is quantified separately. However, the output files do allow for user-driven *post-hoc* quantification. For example, the user can decide to quantify tDRs by aggregating tRNA families (defined here as those tRNAs with identical sequence) into even larger super families based on the isodecoder or the amino acid. This would not allow for per base resolution, but it would simplify the signal.

#### Step 5: Quantified tDRs are visualized as color-coded coverage maps

*tDRmapper* automatically generates a separate visualization of the pre-tRNA-derived tDR profile and mature tRNA-derived tDR profile for each sample. Figure 5 shows one example mature tRNA coverage map from each of the four categories of cell types/tissues. The y-axis of the tRNA coverage map shows the top 50 most highly abundant tDRs in descending order, and also includes the relative abundance (described in Step 4 above). The x-axis shows the position within the tRNA. Each row displays the percent read coverage at each position, the nucleotide sequence of each tRNA, and the positions and proportions of any “error types” that are present at >5 % of the reads that map to a given position. This coverage map enables the user to easily visualize the global tDR profile of a sample.

The coverage maps in Fig. 5 show that tDR profiles can vary dramatically across tissues. For instance, the most abundant type of tDRs in the seminal fluid (Fig. 5b) and liver (Fig. 5c) are of the type tRH-5′, while the colon (Fig. 5a) and neural progenitors (Fig. 5d) have a greater abundance of tRF-3’s. It is worth noting that these differences are not attributable to differences in library preparation or sequencing technology, because the liver and colon libraries were prepared and sequenced using the same protocols in the same sequencing facility.

As discussed above, “error types” serve as a proxy for chemical modifications and therefore can vary across different tissues and diseases (Fig. 3). Our coverage maps annotate the specific “error types” present, enabling comparisons at single nucleotide resolution. For example, for Glu-CTC-1-1, all of the examples shown have a one base deletion at positions 7 or 8 accounting for ~20–30 % of the reads that span that position. However, the liver and seminal fluid samples also have a three base deletion at anywhere between positions 6 and 10 (though at positions 7 and 8 only the one base deletion is shown because it has slightly higher coverage). In humans, position 10 of the tRNA Glu-CTC has a known N2-methylguanosine modification, which may cause reverse transcriptase pausing during the cDNA step, potentially leading to the observed one base or three base deletion at exactly this position [21].

Lastly, it is evident from the coverage maps that the same parental tRNA can produce different primary tDRs depending on the tissue. For example, Gly-GCC-5-1 produces a tRH-DTA in the colon samples, a tRH-5′ in the liver and seminal fluid samples, and a tRF-3′ in the neural progenitor samples (Fig. 5a-d).

**Fig. 6:**
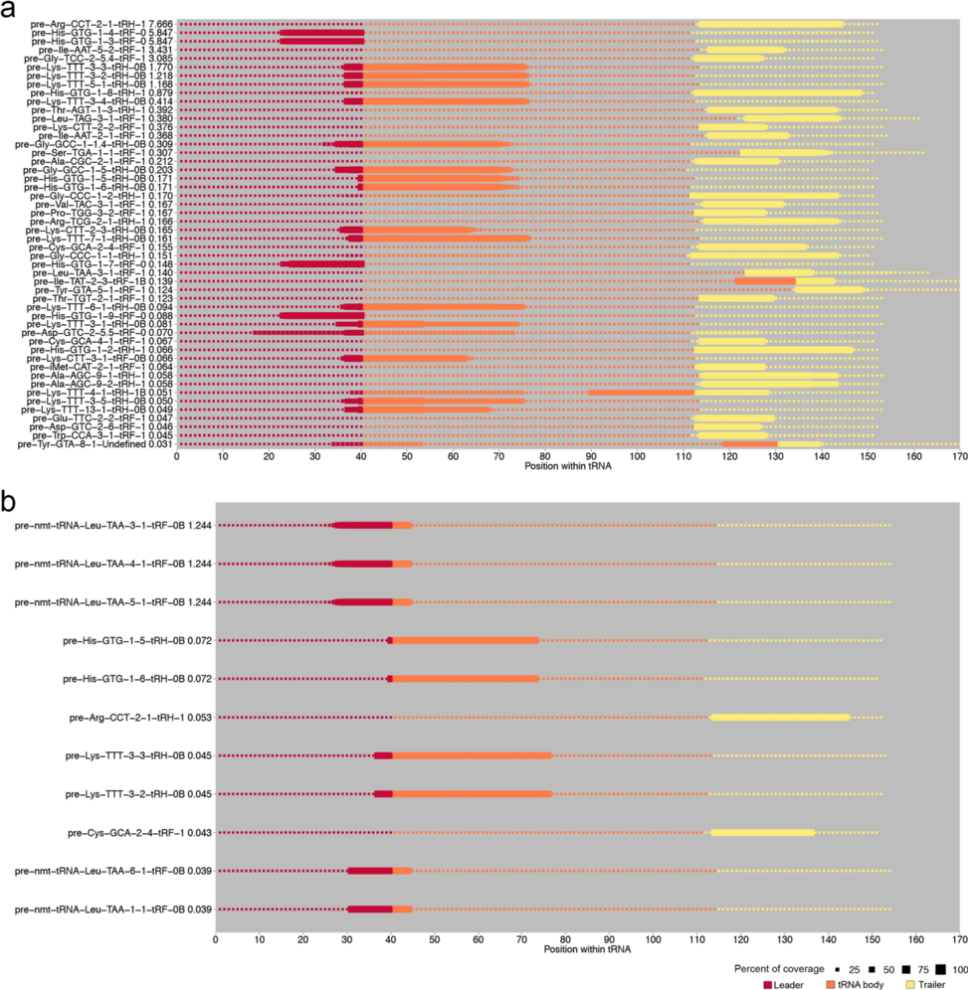
Example of pre-tRNA coverage maps. **a** & **b** Size of dot represents percent of reads mapping at each position within each tRNA. The color represents the location of the pre-tRNA: leader sequence is red, the tRNA body is orange, and the trailer is yellow. Coverage map for (**a**) H1-derived mesendoderm cells and **b** primary tissue from the inflamed section of colon tissue from a subject with Crohn’s disease, #413

Figure 6 shows two pre-tRNA coverage maps. Figure 6a is from an H1-derived mesendoderm sample, which has the highest expression of tDRs derived from pre-tRNAs (pre-tDRs) among all samples analyzed, with 31 % of its tRNA aligned reads mapping to pre-tRNAs. Figure 6b is from an inflamed section of colon tissue from a subject with Crohn’s disease, which has the highest expression of pre-tDRs among the primary tissue/fluid samples analyzed, with 1.2 % of its tRNA aligned reads mapping to pre-tRNAs. These coverage maps are similar to the coverage maps in Fig. 5, but each dot is colored by location in the pre-tRNA instead of each individual base. We do not show each individual base because we are not indicating “error type”, since the algorithm searches only for exact matches to pre-tRNAs. The coverage maps in Fig. 6 shows that reads can be derived from both the leader and trailer sequence, as well as from the region spanning the leader/trailer sequence and the tRNA body.

### Analysis of different categories of cell types/tissues shows that tDR profiles are specific to tissue, disease and stage of differentiation

**Fig. 7:**
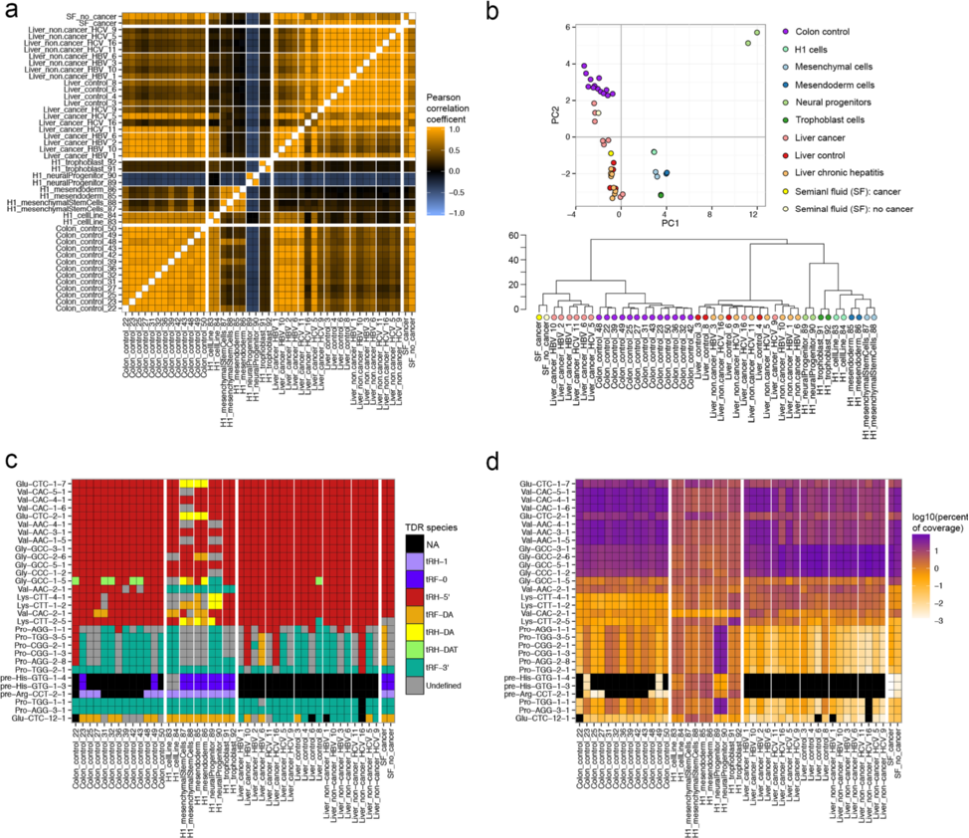
Comparison of tDR profiles across four categories of human cell types/tissues. (A-D) tDR expression profiles for each human cell type/tissue; every tDR that has >5 % relative abundance in any sample is included in the analysis. **a** Pearson correlation coefficient heat map. Each cell in the map represents the correlation coefficient between tDR expression profiles from different two samples. Cells along the diagonal represent identical samples and are colored in white. Thick white lines divide each category of data sets; thin white lines divide sub-categories of datasets within each category. Midpoint of color change for bar r^2^ = 0. **b** *Top panel*: plot of principle components. *Bottom panel*: dendogram from hierarchical clustering of all tDR profiles. **c** Primary tDR species heat map. Each cell represents the dominant tDR species in each sample. **d** tDR expression heat map. Each cell represents the log10 of the relative tDR abundance of each tDR in each sample

Pearson correlation analysis of all pairs of samples across the four categories of datasets shows that tDR profiles correlate strongly between pairs of samples within the same cell type/tissue (median pair-wise r^2^ >0.85, Fig. 7a). This result is indicative of very little variability across individuals (or biological replicates in the case of the stem cells) in tDRs within the same tissue. Generally speaking tDR profiles were far less correlated between pairs of samples across categories; however, interestingly, 6/8 liver cancer samples correlated more strongly with the colon tissue samples (median pair-wise r^2^ = 0.91) than with the non-cancer liver samples (median pair-wise r^2^ = 0.42). The PCA plot shows several of the liver cancer samples closer to the colon samples than the non-cancer liver samples and the hierarchical clustering of the same data revealed that 6/8 liver cancer samples are in the same clade as the colon tissue samples (Fig. 7b), supporting the Pearson correlation analysis (Fig. 7a).

The dominant tDR type produced from the same tRNA family sometimes varies across different samples (Figs. 5 and 7c). For example, the dominant tDR derived from Val-AAC-2-1, which produced a high abundance tDR in all samples, was a tRH-5′ in most colon, liver, and seminal fluid samples but a tRF-3′ in all of the stem and progenitor cells (Fig. 7c and d). Another example is Glu-CTC-1-7, which produces a tRH-5′ in most of the primary tissue/fluid samples, the H1 cells, the trophoblasts, and the neural progenitor samples, but produces a tRH-DA in the mesendoderm and mesenchymal stem cells.

Reads corresponding to pre-tDRs represent 6–31 % of all tDR reads in the stem/progenitor cells, >1 % in two of the colon samples, and <1 % in the rest of the samples. In the H1-derived cells, the most highly expressed pre-tDRs are pre-His-GTG-1-4-tRF-0 and pre-Arg-CTC-2-1-tRH-1, which are tDRs derived from the header and leader sequences, respectively. In the mesendoderm samples, which have the highest pre-tDR abundance among the samples analyzed, a pre-tDR (either pre-His-GTG-1-4-tRF-0 or pre-Arg-CTC-2-1-tRH-1, depending on the sample) is the third-most abundant tDR after Glu-CTC-1-7-tRH-DA and Glu-CTC-2-1-tRH-DA.

The primary tissue/fluid samples are characterized by a few very highly expressed tDRs (Fig. 7d). Specifically, the colon samples and hepatitis B associated liver cancer samples are characterized primarily by tDRs derived from tRNA-Val genes, and the other liver samples by tDRs derived from tRNA-Gly genes. In contrast, the stem and progenitor cells, with the exception of the neural progenitor cells, are not dominated by a few highly expressed tDRs, but rather have many tDRs with moderate expression.

## Conclusions

The biomedical significance of tDRs is becoming increasingly apparent and profiling tDRs from small RNA-seq data sets will define the cell type-specificity of tDRs and their chemical modifications. In this study we introduced a tool for mapping, naming, and quantifying tDRs, called *tDRmapper*, which is designed to handle the unique and challenging features of small RNAs derived from tRNAs. We analyzed small RNA-seq data from four different categories of cell types/tissues using *tDRmapper* and found that tDR profiles can differ dramatically across different tissues and disease states, that RNA modifications (as proxied by “error types”) vary by tissue and disease, and that different tDR species can be derived from the same parental tRNA depending on the tissue. *tDRmapper* not only provides a standardized nomenclature and quantification scheme for tDRs, but also includes graphical visualization that facilitates the discovery of novel tRNA and tDR biology.

## Methods

### tDRmapper package

*tDRmapper* is available on gitHub (https://github.com/sararselitsky/tDRmapper). The folder contains all the scripts needed for *tDRmapper* to run, the human tRNA FASTA file, detailed documentation, and the legend for the coverage maps. *tDRmapper* was written in Perl v.5.12.0 and R v3.1.0. The coverage maps were generated using the ggplot2 R package.

### Defining the tRNA generalized secondary structure

Structures of hg19 tRNAs were downloaded from gtRNAdb. The mode values of the D, anti-codon, and T loops’ start and stop positions across all tRNAs were used to determine the “generalized” location of each loop.

### Figures and statistical analyses

All statistical analyses were conducted using R. The hierarchical clustering was performed using the hclust R package and Ward’s method. The principal components analysis was computed using the R package prcomp. Figures 4, 5, 6, 7 were produced with the R package gglot2.

### Datasets

All the data sets used in this study were publically available and no ethics approval was required for access.

## Abbreviations

tRNA: transfer RNA
tDR: tRNA-derived RNA
bp: Base pair
nt: Nucleotides
small RNA-sequencing: small RNA-seq
